# Low-quality animal by-product streams for the production of PHA-biopolymers: fats, fat/protein-emulsions and materials with high ash content as low-cost feedstocks

**DOI:** 10.1007/s10529-020-03065-y

**Published:** 2020-12-26

**Authors:** Victoria Saad, Björn Gutschmann, Thomas Grimm, Torsten Widmer, Peter Neubauer, Sebastian L. Riedel

**Affiliations:** 1 Chair of Bioprocess Engineering, Institute of Biotechnology, Technische Universität Berlin, Berlin, Germany; 2 ANiMOX GmbH, Berlin, Germany

**Keywords:** Animal by-products, Fat/protein-emulsion, Low-cost feedstocks, *Ralstonia eutropha*, Medium-chain-length PHA, P(HB-*co*-HHx)

## Abstract

**Objective:**

The rapid accumulation of crude-oil based plastics in the environment is posing a fundamental threat to the future of mankind. The biodegradable and bio-based polyhydroxyalkanoates (PHAs) can replace conventional plastics, however, their current production costs are not competitive and therefore prohibiting PHAs from fulfilling their potential.

**Results:**

Different low-quality animal by-products, which were separated by thermal hydrolysis into a fat-, fat/protein-emulsion- and mineral-fat-mixture- (material with high ash content) phase, were successfully screened as carbon sources for the production of PHA. Thereby, *Ralstonia eutropha* Re2058/pCB113 accumulated the short- and medium-chain-length copolymer poly(hydroxybutyrate-*co*-hydroxyhexanoate) [P(HB-*co*-HHx)]. Up to 90 wt% PHA per cell dry weight with HHx-contents of 12–26 mol% were produced in shake flask cultivations.

**Conclusion:**

In future, the PHA production cost could be lowered by using the described animal by-product streams as feedstock.

**Graphical abstract:**

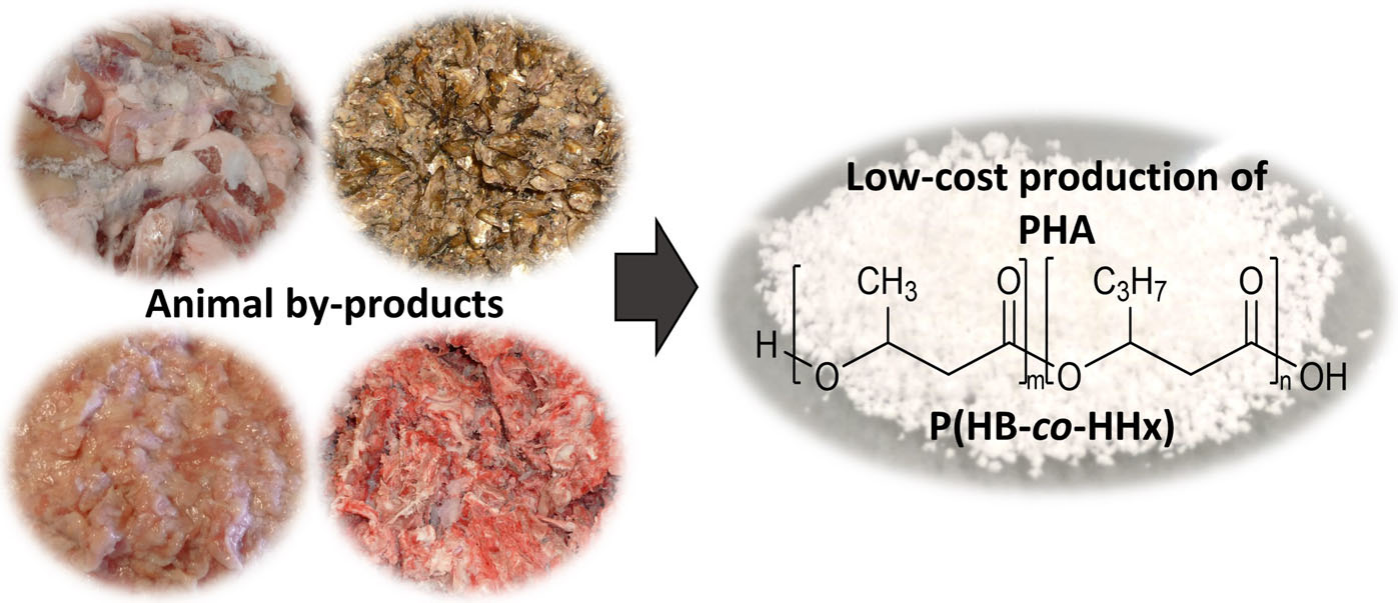

## Introduction

Within the last decade, more crude-oil-based plastic was produced than during the whole twentieth century. The resulting environmental pollution with (micro-)plastic (Lambert and Wagner 2018), due to insufficient recycling schemes as well as the required finite crude-oil-resources are reasons to increase the use of so-called bio-based and biodegradable bioplastics such as polyhydroxyalkanoates (PHAs). Currently, their market share only accounts for 1.2% of the bioplastics production capacity (European Bioplastics 2019), mostly due to their comparatively high market price. Feedstock and downstream processing are primarily responsible for the costs and the reason why PHAs play a minor role in industrial applications (Gahlawat 2019; Riedel and Brigham 2020).

Every year, the meat industry generates around 100 million tons of animal by-products, e.g. up to 30% of the live weight from a pig and up to 40% of the live weight from cattle end up as animal by-products (Mora et al. 2019). Animal by-products are classified into three classes by the European Union (Official Journal of the European Union 2009): Class 1 materials are mostly coming from infectious animals and need to get burned. Materials from not slaughtered animals as e. g. perished animals belonging to Class 2 and all animal by-products from slaughter, cutting and meat processing, which for economic and/or cultural reasons are not intended for human consumption belong to Class 3. Low-quality animal by-products are often wasted, burned or used for biogas production (Kapoor et al. 2020; Lecrenier et al. 2020). However, depending on their class and quality they can be used as a low-cost substrate for the production of food ingredients, enzymes, pet food, fertilizer, pharmaceuticals and other value-added products (Toldrá et al. 2016). Nevertheless, the potential of animal by-products as substrates for PHA production has only been studied by a few groups as reviewed by (Koller et al. 2018). The company ANiMOX GmbH invented a process in which animal by-products can be efficiently separated by thermal hydrolysis into a fat-, protein/water- (protein hydrolysate), fat/protein-emulsion- and mineral-fat-mixture- (material with high ash content; bone sediment) phase (Höhling et al. 2006). So far, only the separated fat phase has been studied as potential feedstock for PHA-production (Riedel et al. 2015). But especially the low-quality fat/protein-emulsions, fat-greaves and minerals-fat-mixtures have even lower market prices and therefore the potential to lower PHA-feedstock costs further.

The Gram-negative betaproteobacterium *Ralstonia eutropha* is the model organism for PHA production since it can consume a variety of carbon sources (e.g. consumption of lipids is enabled through secretion of an extracellular lipase), can accumulate large amounts of PHA (up to 90 wt%), can be grown to high cell densities (> 250 g L^−1^) and tools for genetic engineering are readily available (Ryu et al. 1997; Reinecke and Steinbüchel 2008; Riedel et al. 2014; Brigham and Riedel 2018; Raberg et al. 2018).

PHAs show biodegradable behavior in all aerobic and anaerobic environments defined by ASTM standards (Meereboer et al. 2020). While the most common short chain length PHA polymer, polyhydroxybutyrate (PHB), is difficult to process among others due to its brittleness, the short- and medium chain length PHA-heteropolymer poly(hydroxybutyrate-*co*-hydroxyhexanoate) [P(HB-*co*-HHx)] has polypropylene-like properties, which can be tuned through the HHx-content (Noda et al. 2005). In this study, low-quality animal by-product streams were initially evaluated in shake flask experiments as potential feedstocks to produce P(HB-*co*-HHx) with *R. eutropha* Re2058/pCB113.

## Materials and methods

### Bacterial strain

Throughout this study, the engineered *R. eutropha* strain Re2058/pCB113 was used to produce P(HB-*co*-HHx) (Budde et al. 2011).

### Animal by-product streams

**Fig. 1:**
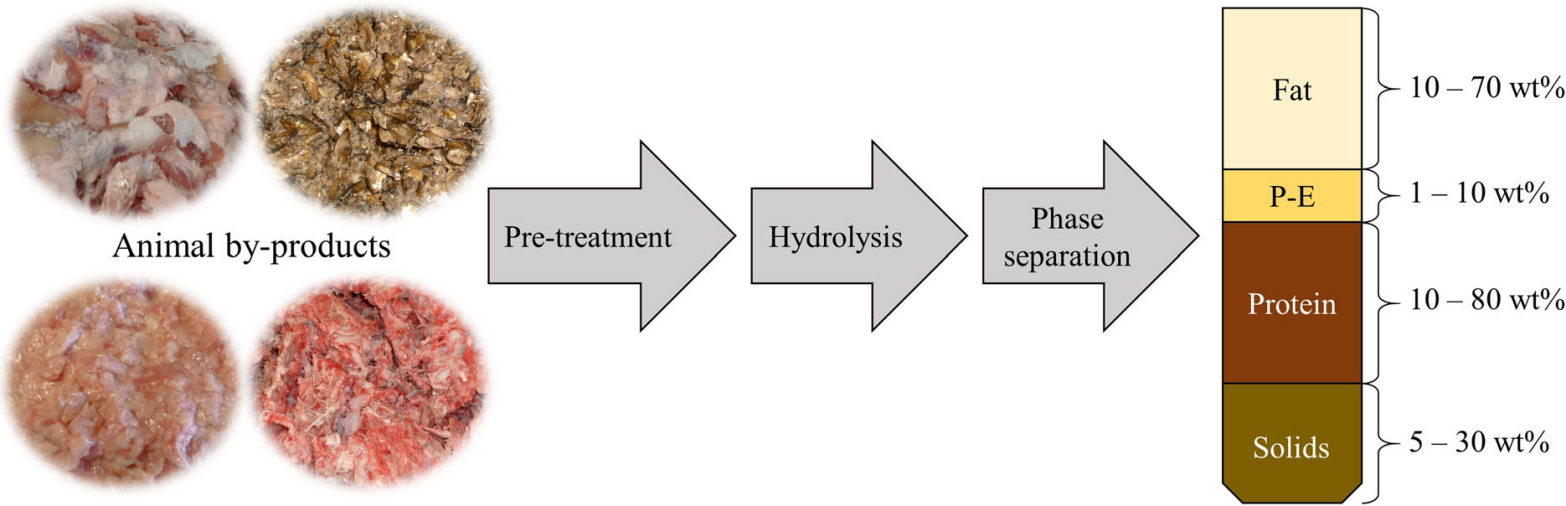
Production of separated fats (Fat), fat/protein-emulsion (P–E), protein hydrolysate (Protein) and mineral-fat-mixture (Solids) or fat-greaves (Solids) from animal by-products via thermo-pressure hydrolysis after mechanical or enzymatical pre-treatment (Höhling et al. 2006)

The animal by-products of Class 3 were provided by ANiMOX GmbH (Berlin, Germany) and are the result of thermo-pressure hydrolysis at temperatures in the range of 130–180 °C, a pressure of 3–20 bar with an incubation time of 1–120 min, after mechanical or enzymatical pre-treatment (Table 1, Fig. 1). While the enzymatic hydrolysis of proteins only hydrolyzes molecules that are attackable by enzymes, the thermo-pressure hydrolysis destroys the entire material structure including bones and releases molecules such as fat and proteins. The animal by-products either have low-fat contents (e.g. left-over bones at a butcher) or are highly processed (e.g. head and tails from smoked sprats or fat/protein-emulsions and mineral-fat-mixtures).

**Table 1:** Overview of used animal by-product streams

Group	Origin	Content	Term
Separated fats	Fish	Fish fat (from smoked sprats)	ANiFAT F
	Pork	Pork fat	ANiFAT P
Technical fractions with mixed-fat-parts	Pork	Fat/protein-emulsion	ANiFAT P-E
	Pork	Mineral-fat-mixture (with high ash content)	ANiFAT P-S
	Pork	Fat-greaves	ANiFAT P-G

### Cultivation conditions

#### Growth media

Tryptic soy broth (TSB) media was used for the first preculture and with an additional supply of 2 wt% agar for culture plates. The second preculture and shake flask cultivations were conducted in phosphate-buffered mineral salt media (Gutschmann et al. 2019). As a carbon source, animal by-product streams or canola oil were used in different concentrations as described in the text. Urea in the concentration of 0.28 g L^−1^ was used as nitrogen source in all cultivations. The carbon source was autoclaved together with the phosphate buffer, K_2_SO_4_ and NaOH in the shaking flasks for 20 min at 121 °C. The other media components were added from sterile stocks before inoculation. Chemicals reagents were purchased from Sigma Aldrich (USA), Carl Roth GmbH & Co. KG (Germany), Merck KGaA (Germany) or VWR Chemicals (USA).

#### Shake flask experiments

*R. eutropha* Re2058/pCB113 was streaked on a TSB agar plate from a cryoculture and incubated for 3–4 days at 30 °C. The first preculture in 10 mL TSB media in a 125-mL Ultra Yield flask (Thomson Instrument Company, USA), sealed with an AirOtop membrane (Thomson Instrument Company, USA), was inoculated with a single colony from the plate. After 14 h of incubation an optical density (OD_600_) of 4–5 was reached and 0.5 mL of the culture were used to inoculate the second preculture (50 mL MSM containing 1 wt% canola oil) in a 250-mL DURAN baffled glass flask (DWK Life Sciences GmbH, Germany) sealed with an AirOtop membrane. After 24 h of incubation, 0.5 mL of the seed culture were inoculated into a 250-mL DURAN baffled glass flask with 50 mL mineral salt media containing 1–2 wt% of the animal by-product. Each condition was tested in triplicates and all cultures were incubated at 30 °C and shaken at 200 rpm in an orbital shaker with 25 mm amplitude (INFORS HT Multitron Standard, Infors AG, Switzerland).

### Analytical methods

#### Fatty acid distribution of animal by-product streams

For determination of the fatty acid distribution 3–150 mg of the animal by-product were weighed in glass tubes with PTFE-coated caps. The samples were resuspended in 2 mL *n*-hexane and transesterified by acidic methanolysis using dried methanol and acetyl chloride. Nonadecanoic acid was used as internal standard. After acidic methanolysis the fatty acid methyl esters (FAMEs) were extracted with chloroform, which was subsequently evaporated in a rotary evaporator. The residue was resuspended in 2 mL of *n*-hexane prior to GC analysis. The samples were analyzed with a capillary column (WCOT FUSED SILICA 25 m × 0.25 mm ID, DF = 0.12 μm COATING CP-Sil 5 CB, Varian, Germany). For separation of lipids, the following temperature program was set: The starting temperature of 150 °C was maintained for 2 min before the temperature increased by 15 °C min^−1^ to a temperature of 250 °C, holding 250 °C for 30 min. Then, temperature increased by 5 °C min^−1^ to 280 °C, holding 280 °C for 7 min. The injector and detector temperature were set at 290 °C and 300 °C, respectively. The samples were injected in a splitless mode with an autosampler AOC-20i. The injected sample volume was 0.3 μL.

#### Physical and chemical properties of the animal by-product streams

The melting temperatures (T_m_) of the different feedstocks were determined through stepwise heating 1 g of each sample in a glass tube until completely liquefied in a thermomixer (Hettich Benelux, Netherlands). Protein, nitrogen, and ash contents were analyzed by Animox GmbH as described previously (Riedel et al. 2015).

#### Cell dry weight determination

10 mL samples of the shake flask content were collected after 72 h in pre-weighed 15-mL polypropylene tubes. Cells were harvested by centrifugation (6800×*g*, 10 min at 4 °C) and washed with a mixture of 2 mL pre-cooled *n*-hexane and 5 mL de-ionized water to remove residual lipids from the samples. The pellet was resuspended in 2 mL de-ionized water, frozen at − 80 °C, and subsequently lyophilized to obtain the cell dry weight (CDW). Prior to sampling, the final culture volume was determined to include culture concentration due to water evaporation during CDW determination while considering the theoretical culture volume (50 mL) (Eq. 1).

1 $$ {\text{CDW}} \left[ {{\text{g}} {\text{L}}^{ - 1} } \right] = \frac{{{\text{Dry}}\;{\text{weight}}\;{\text{of}}\;{\text{sample}}\;\left[ {\text{g}} \right]}}{{{\text{Sample}}\;{\text{volume}}\;\left[ {\text{L}} \right]}}*\frac{{{\text{Determined}}\;{\text{culture}}\;{\text{volume}}\;\left[ {\text{L}} \right]}}{{{\text{Theoretical}}\;{\text{culture}}\;{\text{volume}}\;\left[ {\text{L}} \right]}} $$

#### Polyhydroxyalkanoate quantification

To quantify the amount and determine the composition of the intracellular PHA in the cells, 10–20 mg of freeze-dried cells were weighed and placed in glass tubes with PTFE-coated caps. The samples were transesterified by acidic methanolysis. Analysis was performed with a gas chromatograph (GC-2010 Plus, Shimadzu Corp., Kyoto, Japan) as described previously (Bartels et al. 2020).

## Results

### Feedstock characterization

Prior to cultivation, the analysis of physical and chemical properties (Table 2) and fatty acid compositions (Table 3) was conducted. The animal by-products had a large variation in the dry matter (49–97 wt%) total fat content (4–96 wt%), free fatty acid (FFA) content (4–52%), total protein concentration (1–18 wt%), nitrogen content (0.1–2.8 wt%), ash content (0–35 wt%) and melting temperature, respectively. Some animal by-products could not be melted, due to their higher protein content.

**Table 2:** Physical and chemical data of the animal by-product streams (ANiFATs) and canola oil

Feedstock	Dry matter (wt%)	Total fat (wt%)	FFA^a^ (%)	Total N (wt%)	Total protein (wt%)	Ash (wt%)	T_m_ (°C)
Canola oil	100	100	N/A	0.0	0.0	0.0	< RT
ANiFAT F	97.4	95.8	5.5	0.2	1.3	0.2	< RT
ANiFAT P	90.4	89.8	51.8	0.5	3.1	0.0	50
ANiFAT P-E	52.8	37.9	49.0	1.8	11.1	2.4	–^b^
ANiFAT P-G	54.4	36.1	1.9	2.8	17.7	0.6	–^b^
ANiFAT P-S	49.0	4.21	3.6	1.4	8.9	35.0	< RT^c^

*T*_*m*_ Melting temperature, *N/A* data not available, *FFA* free fatty acids, *RT* room temperature (24 °C)

^a^Of total fat content

^b^No melting takes place due to the high protein content

^c^The bone sediment particles stay solid

**Table 3:** Fatty acid distribution (wt%) of the feedstocks determined in triplicates

Feedstock	C14:0	C16:0	C16:1	C18:0	C18:1–3	C20:0	C20:3	C22:2
CO	0.09 ± 0.55	4.2 ± 9.2	0.5 ± 0.8	2.0 ± 0.0	89.5 ± 20.4	0.21 ± 0.05	1.18 ± 0.14	0.44 ± 0.05
AF-F	5.70 ± 0.02	26.8 ± 0.7	9.2 ± 0.0	4.5 ± 0.0	47.7 ± 2.0	0.47 ± 0.01	0.43 ± 0.12	0.48 ± 0.01
AF-P	1.20 ± 0.00	19.5 ± 0.1	2.5 ± 0.0	14.1 ± 0.0	47.6 ± 0.1	0.22 ± 0.02	0.39 ± 0.02	0.18 ± 0.02
AF-P-E	0.98 ± 0.10	22.1 ± 4.0	1.5 ± 0.3	19.2 ± 0.0	36.1 ± 6.7	0.09 ± 0.06	0.51 ± 0.08	0.27 ± 0.01
AF-P-G	0.95 ± 0.03	20.0 ± 0.7	1.7 ± 0.1	16.0 ± 0.0	44.7 ± 2.0	0.11 ± 0.01	0.44 ± 0.12	0.13 ± 0.01
AF-P-S	0.86 ± 0.03	20.1 ± 0.5	2.2 ± 0.1	14.8 ± 0.0	46.7 ± 1.5	0.14 ± 0.01	0.32 ± 0.02	0.19 ± 0.01

CO: canola oil, AF: ANiFAT. ± indicates standard deviation from triplicate measurements

Since it has been demonstrated that the fatty acid composition of the carbon source may affect the HHx-content of the produced polymer (Riedel et al. 2012; Wong et al. 2012), initially the composition of the different animal by-product streams was analyzed. The fatty acid compositions of all feedstocks (Table 3) were similar, the most prevalent chain lengths were C14–C18. While canola oil is mostly based on the unsaturated C18:1–3 (91 wt%), only approximately 50 wt% of the animal by-product streams belonged to these unsaturated fatty acids. Instead, C16:0 and C18:0 both made up about 25 wt% and 20 wt% respectively. Traces of C12:0, C14:0, C16:1, C20:0, C20:3, C22:0 and C22:2 were found in all feedstocks.

### Growth and PHA production evaluation in shake flask experiments

**Fig. 2:**
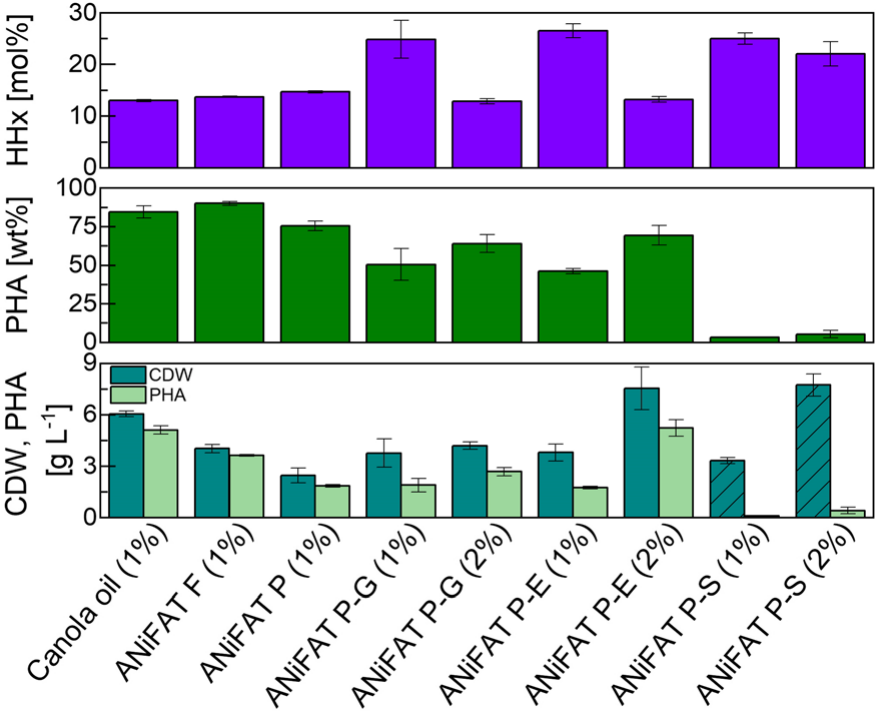
Cell dry weight (CDW, g L^−1^), polyhydroxyalknaoate (PHA, g L^−1^), PHA content per CDW (wt%) and HHx-content per PHA (mol%), after 72 h of shake flask cultivation with *R. eutropha* Re2058/pCB113 grown on canola oil or different animal by-product streams (1–2 wt%) in mineral salt media. Striped lines indicate that the biomass pellet also contained residual solids from the used feedstock. Error bars indicate standard deviation from triplicate cultivations

Shake flask cultivations with *R. eutropha* Re2058/pCB113 showed that the cells grew on all animal by-product streams (Fig. 2). The engineered strain was able to naturally emulsify all feedstocks in the shake flask cultivations. The maximal CDW (7.6 ± 1.2 g L^−1^) was reached with 2 wt% fat/protein-emulsion (ANiFAT P-E). In cultivations with 2 wt% mineral-fat-mixture (ANiFAT P-S) the determined CDW was higher, however, the biomass pellet also contained residual solids from the feedstock and can therefore not be taken into account. Nevertheless, an increase in PHA production from 0.1 to 0.4 g L^−1^ was obtained when increasing the mineral-fat-mixture concentration from 1 to 2 wt%, indicating that cell growth and PHA accumulation occurred. Using 1 wt% canola oil resulted in a CDW of 6.1 ± 0.2 g L^−1^. The lowest biomass (2.5 ± 0.4 g L^−1^) was obtained with pork fat (ANiFAT P), which has a very high free fatty acid content (52%). PHA was accumulated under all cultivation conditions. *R. eutropha* Re2058/pCB113 contained up to 90 wt% of PHA when grown on 1 wt% fish fat (ANiFAT F).

Generally, 1 wt% canola oil, fish or pork fat was enough to yield relevant CDW and PHA values for the applied nitrogen concentration. Due to the lower fat content of the fat/protein-emulsion (ANiFAT P-E) and fat-greaves (ANiFAT P-G) comparable results were able to be achieved by doubling the feedstock concentration to 2 wt%.It is well known that the HHx-content decreases with increasing PHA accumulation in the used engineered strain, ending with a HHx-content of 13–17 mol% in shake flask cultivations with similar composed feedstocks (Riedel et al. 2014, 2015). Subsequently, in the shake flask cultivations, the highest HHx-content of 26.5 mol% (46 wt% PHA per CDW) was observed in a cultivation with 1 wt% fat/protein-emulsion (ANiFAT P-E). The HHx-content decreased to 13 mol% when doubling fat/protein-emulsion concentration, at the same time the PHA content per CDW could be increased to 69 wt%.

## Discussion

The general potential of industrially obtained low-quality animal by-product streams (fat, fat/protein-emulsion, fat-greaves and mineral-fat-mixture with high ash content) as suitable carbon sources for *R.* *eutropha* was demonstrated in shake flask cultivations. The final total biomass reached with separated pork fat was the lowest (Fig. 2). According to (Nieman 1954; Hogan et al. 1987) it is probable that interaction between the cell surface and the free fatty acids of the separated pork fat occurs, thus leading to altered cell permeability and resulting in inhibition of cell growth (Greenway and Dyke 1979). Interestingly, however, no growth-inhibiting effects were observed in cultivations with the pork-based fat/protein-emulsion, which also had a very high free fatty acid content of 49% (Table 2). The emulsion with the proteins must prevent the growth-inhibiting effect of the free fatty acids. The highest PHA content per CDW was reached with canola oil and fish fat (Fig. 2), which contained no or low nitrogen, respectively (Table 2). Since the PHA-synthesis was triggered with nitrogen limitation in this study, the presence of nitrogen sources in the biogenic feedstock probably affected the biomass and PHA formation by changing the C/N ratio. The highest biomass was reached with the 2 wt% pork-based fat/protein-emulsion cultivations, which had less carbon content than the 1 wt% canola oil cultivations but contained additional nitrogen from residual proteins. However, the 2 wt% pork-based fat-greaves cultivations reached significantly less biomass (4.21 g L^−1^) than the 2 wt% pork-based fat/protein-emulsion cultivations (7.55 g L^−1^) but contained 56% more nitrogen and only 5% less fat (Table 2). Therefore, the ideal C/N ratio for each feedstock must be investigated during further bioprocess development. Additionally, the occurring protein hydrolysates (Fig. 1) could be used as an inexpensive complex nitrogen source, as it has been previously described by using hydrolysate from chicken feather (Benesova et al. 2017). However, depending on the protein content, the animal by-products could maybe serve as complex carbon and nitrogen source.

Also, further bioprocess development in bioreactors is necessary to find out whether the animal by-product streams not only meet the criteria leading to a PHA enrichment of > 50 wt% per cell but also meet other criteria such as supporting high productivity and total PHA production, which are described in the literature (Koller et al. 2017; Riedel and Brigham 2019; Zheng et al. 2020) as prerequisites for successful PHA production. Therefore, distinct feeding strategies must be developed for each animal by-product stream. As some animal by-product streams cannot be thermally liquefied (fat/protein-emulsion and the fat-greaves) or contain solids (mineral-fat mixtures), feeding via e.g. a screw conveyor must be implemented. A laboratory method for separating the residual minerals from the biomass must be established in order to quantify the correct CDW and PHA content of the cells. The recently described *in-line* method for *real-time* quantification of PHA accumulation and biomass formation (Gutschmann et al. 2019), could be used for process control during bioreactor cultivations, especially when an exact *off-line* quantification of both is only hardly possible. In addition, the impact of the residual minerals in the harvested biomass on downstream processing needs to be assessed, as separating the minerals before PHA recovery would increase the overall process costs. Furthermore, the mixed animal by-product streams have a lower carbon content than the separated fat fractions. Therefore, more material is needed to achieve a sufficient yield, which makes the transport of raw materials more expensive compared to the separated animal fats. To keep transport costs as low as possible, the establishment of decentralized PHA factories next to animal processing plants would be recommended for the realization of such production facilities.

Nevertheless, all tested animal by-product streams show great potential to be used as feedstock for cost-effective PHA production. They also avoid the "food vs. commodity" controversy. Also, the low-quality animal by-product streams studied have the advantage that they are not or not well suitable for other industries as e.g. the biodiesel industry, due to their very high content of free fatty acids and/or proteins, ash and low total fat content (Toldrá-Reig et al. 2020).

## Conclusion

Fat/protein-emulsions, mineral-fat-mixtures and fat-greaves, solid products from the thermo-pressure hydrolysis of animal by-products were used for the first time to produce PHA. Nitrogen from residual proteins in the animal by-products influenced biomass and PHA formation. Growth inhibition caused by a high content of free fatty acids (~ 50%) in fat cultivations did not occur in fat/protein-emulsion cultivations. Further bioprocess development in bioreactors with an adapted C/N ratio and a feeding method for the non-meltable animal by-products is necessary for an efficient low-cost PHA production.
